# Tyrosine kinase inhibition to improve anthracycline-based chemotherapy efficacy in T-cell lymphoma

**DOI:** 10.1038/s41416-019-0557-8

**Published:** 2019-09-02

**Authors:** Martina Magni, Giulia Biancon, Sara Rizzitano, Alessandra Cavanè, Chiara Paolizzi, Matteo Dugo, Paolo Corradini, Cristiana Carniti

**Affiliations:** 1 Department of Medical Oncology and Hematology, Fondazione IRCCS Istituto Nazionale dei Tumori, Milan, Italy; 2 Department of Oncology and Hemato-oncology, Università degli Studi di Milano, Milan, Italy; 3 Department of Applied Research and Technological Development, Fondazione IRCCS Istituto Nazionale dei Tumori, Milan, Italy; 4 Present Address: Yale University School of Medicine, Comprehensive Cancer Center, Hematology, New Haven, CT 06511 USA

**Keywords:** Non-hodgkin lymphoma, Molecular medicine

## Abstract

**Background:**

Anthracycline-containing regimens, namely cyclophosphamide, doxorubicin, vincristine and prednisone (CHOP) or CHOEP (CHOP + etoposide), represent the current standard of care for patients with newly diagnosed peripheral T-cell lymphomas (PTCLs), although responses are unsatisfactory. In this study, we investigated the pathways able to mitigate the sensitivity to CHOP-based regimens in preclinical models of T-cell lymphoma (TCL) to select agents for the development of CHOP-based drug combinations.

**Methods:**

We performed gene expression profiling of malignant T-cell lines exposed to CHOEP; flow cytometry was employed to study the effects of drug combinations on cell viability, cell cycle distribution, apoptosis and mitochondrial membrane depolarisation. Western blot analyses were performed to study cell signalling downstream of the T-cell receptor and apoptosis. The in vivo effect of the drug combination was tested in xenograft models.

**Results:**

We highlighted a modulation of tyrosine kinases belonging to the T-cell receptor pathway upon chemotherapy that provided the rationale for combining the tyrosine kinase inhibitor dasatinib with CHOEP. Dasatinib improves CHOEP activity and reduces viability in vitro. Furthermore, combination treatment results in tumour growth inhibition in in vivo xenograft mouse models.

**Conclusions:**

Our data provide the rationale for clinical testing of the dasatinib–CHOEP combination in patients with T-cell lymphoma.

## Background

Mature T-cell lymphomas (TCLs) are a phenotypically heterogeneous group of malignancies representing 10–15% of non-Hodgkin lymphomas (NHL). They comprise different cutaneous, extranodal and nodal entities that are often referred to as peripheral T-cell lymphomas (PTCL) to distinguish them from immature T-cell neoplasms.^1^ Nodal forms are the most common and include well-characterised histotypes, such as angioimmunoblastic T-cell lymphoma (AITL) and anaplastic large T-cell lymphoma (ALCL, ALK + or ALK−). Nonetheless, ~30% of PTCL cases fail to be classified into the known subtypes and, based on exclusion criteria, they are categorised as PTCL not otherwise specified (PTCL-NOS).^2^ Cases of PTCL tend to have an aggressive clinical course, with poor responses to conventional chemotherapy and poor long-term survival.^3^ Anthracycline-based chemotherapy programs, predominantly cyclophosphamide, doxorubicin, vincristine and prednisone (CHOP) or CHOEP (CHOP + etoposide), are widely used in the upfront treatment of PTCLs, despite suboptimal results.^4–6^ Neither intensified/escalated chemotherapeutic approaches^7,8^ nor the addition of monoclonal antibodies^9^ have demonstrated a clear advantage in response rates and survival.

We and others have recently demonstrated that the achievement of complete response (CR) before stem cell transplantation is a prerequisite for long-term disease control.^10^ However, 25–30% of patients do not become transplant eligible due to primary refractory or early progressive disease, have very poor prognosis with few therapeutic options. Alternative approaches as well as new molecular druggable targets need to be identified to induce CR after first-line therapy. We speculated that the identification of pathways able to mitigate the sensitivity to CHOP-based regimens, might help selecting agents to develop rational CHOP-based drug combinations to be rapidly translated to the clinical setting.

To address this issue, we performed gene and protein expression analyses in a panel of malignant T-cell lines treated with CHOEP and we found that chemotherapy modulates the T-cell receptor (TCR) pathway, resulting in enhanced expression of genes encoding for pro-survival proteins and the upregulation of tyrosine kinase activity. In line with this finding, we found a cooperative effect when the tyrosine kinase inhibitor (TKI) dasatinib^11^ was combined with CHOEP in vitro and in vivo. Given dasatinib efficacy and tolerability when administered for the treatment of chronic myeloid leukaemia (CML) and acute lymphoblastic leukaemia (ALL),^12^ our results suggest that dasatinib could be used in combination with anthracycline-based programs in the upfront treatment of TCLs to improve outcome.

## Methods

### Cell lines and treatments

HH, HD-MAR-2, JURKAT, SUP-T1 and KARPAS-299 were obtained from the German Collection of Microorganisms and Cell Cultures (DSMZ, Braunschweig, Germany). OCI-Ly12 cell line was a generous gift from Dr Leandro Cerchietti (Weill Cornell Medicine, NY, USA as approved by the Ontario Cancer Institute). Cells were grown in a humidified chamber with 5% CO_2_ at 37 °C, in the RPMI 1640 medium (Lonza, Basel, Switzerland) supplemented with 10% FBS, 1% L-glutamine and 100 U/ml pen/strep. Healthy T lymphocytes were obtained from the peripheral blood of healthy volunteer donors isolated upon density-gradient centrifugation and separation with the autoMACS Pro separator (Miltenyi Biotec, Bergisch Gladbach, Germany) using CD3 Micro Beads (Miltenyi Biotec) following the manufacturer’s instructions. CHOP was prepared as described in ref. ^13^: cyclophosphamide monohydrate 5.84 pM (C), doxorubicin hydrochloride 1.5 pM (H), vincristine sulfate 260 pM (O) and prednisone 1 µM (P). All drugs, including dasatinib, sorafenib and ruxolitinib, were obtained from Selleck Chemicals (Houston, TX, USA).

JURKAT cells resistant to CHOEP were established by culturing the JURKAT cell line in the appropriate medium supplemented with increasing concentrations of CHOEP (starting from IC_20_ to fivefold IC_50_). The dose was increased after at least three passages. Simultaneously, the parental cell line was grown without treatment to preserve the sensitivity to CHOEP. Experiments reported were performed using cells resistant to twofold IC_50_.

### Cell viability, cell cycle, cell death and measurement of the mitochondrial transmembrane potential

To test cell viability, cells were plated at a density of 5 × 10^4^ viable cells/ml and treated. Upon incubation, cells were labelled with propidium iodide (PI) solution (2 µg/ml) (Millipore Sigma, Darmstadt, Germany) and the count of viable cells (PI negative) was performed using the flow cytometer MACSQuant Analyzer (Miltenyi Biotec).

For cell cycle analyses, cells were fixed in 70% ethanol and stained with 2 µg/ml PI. Cell cycle distribution was measured by flow cytometry (MACSQuant Analyzer).

For assessment of apoptosis cells were stained with Annexin V and PI (Annexin V-FITC Kit, Miltenyi Biotec) following the manufacturer’s instructions and analysed by flow cytometry (MACSQuant Analyzer). Mitochondrial membrane depolarisation was assayed using the fluorescent probe tetramethylrhodamine ethyl ester-TMRE (Thermo Fisher Scientific, Waltham, MA, USA) following the manufacturer’s instructions and measured by flow cytometry (MACSQuant Analyzer).

All data were analysed using the MACSQuantify software version 2.6 (Miltenyi Biotec).

### Gene expression profiling

HH, HD-MAR-2, JURKAT, SUP-T1 and KARPAS-299 cells were exposed to IC_50_ CHOEP (CHOP + etoposide) for 24 h. Time and dose were selected to better appreciate possible modulations of genes’ expression. Upon harvesting, RNA was extracted using the RNeasy Mini kit (Qiagen, Hilden, Germany), quantified using Qubit 2.0 (Thermo Fisher Scientific) and assessed for integrity with Bioanalyzer 2100 (Agilent, Santa Clara, CA, USA). Biotinylated complementary RNA (Illumina TotalPrep RNA Amplification Kit, Thermo Fisher Scientific) was hybridised to HumanHT-12 v4 Expression BeadChips (Illumina, San Diego, CA, USA), and raw data were retrieved using Illumina BeadStudio software version 3.8. The data were then processed using R/Bioconductor software (version 2.13). The *lumi* package^14^ was employed for robust spline normalisation and log2 transformation. The *limma* package^15^ was used for differential expression analysis. Genes with a fold change (FC) ≤ −2 or ≥ 2 and a Benjamini–Hochberg false discovery rate (FDR) < 0.05 were selected as differentially expressed. Functional annotation of differentially expressed genes was performed using DAVID Bioinformatics Resources version 6.8^16,17^ (https://david.ncifcrf.gov). An enrichment *p*-value < 0.05 was considered statistically significant. Gene expression data have been deposited in the NCBI Gene Expression Omnibus database, accession number GSE129289.

### Western blotting

Cells were lysed with Cell Lysis Buffer (Cell Signaling Technology, Danvers, MA, USA) following the manufacturer’s instructions. Proteins were quantified using the Qubit 2.0 fluorimeter and the Qubit Protein Assay kit (Thermo Fisher Scientific). Equal amounts of proteins were subjected to gel electrophoresis using the NuPAGE system (Thermo Fisher Scientific). Proteins were transferred to a nitrocellulose membrane, which was blocked with 4% western milk in TBS-TWEEN 0.1% to saturate the non-specific sites. Incubation with primary antibodies was performed overnight at 4 °C, following the manufacturer’s instructions. Antibodies used were phospho-tyrosine (P-Tyr-100) #9411, phospho-SRC family (Tyr416) #2101, SRC family #2108, phospho-AKT (Ser473) #9271, phospho-ERK1/2 (Thr202/Tyr204) #9101, cleaved caspase 3 (Asp175) #9661; cleaved caspase 9 (Asp315) #9505 (Cell Signaling Technology), β-actin #A2066, Vinculin #V9131 (Millipore Sigma). Proteins were visualised with ECL reagent (GE Healthcare, Little Chalfont, UK).

### Phospho-kinase proteome profiler array

Proteome Profiler Human Phospho-Kinase Array Kit (R&D Systems, Minneapolis, MN, USA) was used following the manufacturer’s instructions. Briefly, the array membranes (A and B), spotted with duplicates of antibodies which recognise different phospho-proteins, were incubated with 500 μg of cell lysates. A cocktail of biotinylated antibodies was added, then streptavidin–HRP incubation was performed. Chemiluminescent detection reagents provided were used to reveal the amount of phosphorylated protein bound to the membrane. Signals detected were quantified using the open source software ImageJ. The signal of negative control was subtracted from each spot, and values were normalised on positive control present in each membrane. Then, a ratio of signal intensity (treated/untreated) was calculated.

### *FYN* and *RHOA* amplification

Genomic DNA was extracted from TCL cell lines using the Nucleospin Tissue kit (Macherey-Nagel GmbH & Co, Düren, Germany) and quantified using Qubit 2.0 and the Qubit DNA HS Assay kit (Thermo Fisher Scientific). *RHOA* and *FYN* sequences containing the nucleotides of interest were amplified by PCR using the following primers: RHOA_for GCCCCATGGTTACCAAAGCA; RHOA_rev GCTTTCCATCCACCTCGATA; FYN-SH2_for ACAGGACTCCACTCACAAGG; FYN-SH2_rev ACTTGGCCGAAAAGATGCTG; FYN-CT_for TGAGCTCATGATCCACTGCT; FYN-CT_rev CTGGCTACGGAATTGAAAGC. FYN-SH2 amplicons contain the coding sequences of Leu174 and Arg176, FYN-CT amplicons of Tyr531. Oligonucleotides were purchased from Metabion (Planegg-Steinkirchen, Germany). Amplified DNA was subjected to Sanger sequencing to verify the presence of missense mutations.

### Animal studies

15 × 10^7^ cells (HD-MAR-2 or OCI-Ly12) in 50% Matrigel (BD Biosciences, San Jose, CA, USA) were subcutaneously injected into the flanks of 5- to 7-week-old NOD/SCID mice (Charles River, Wilmington, MA, USA). Treatment was initiated when tumour volume reached 150–200 mm^3^.

Mice were divided into four cohorts of 8–10 mice per cohort and treated as follows: (i) control cohort (ctrl): 0.2 ml of saline i.v. e 0.2 ml of H_2_O per OS. (ii) Dasatinib (DA) cohort: 30 mg/kg per OS, 5 days for 2 weeks. (iii) CHOEP cohort: one 5-days single cycle of CHOEP. Cyclophosphamide day 1, 40 mg/kg i.v.; doxorubicin day 1, 3.3 mg/kg i.v.; vincristine day 1, 0.5 mg/kg i.v.; prednisone from day 1 to day 5, 0.2 mg/kg per OS; etoposide day 1 and day 3, 3.3 mg/kg i.v. (iv) DA + CHOEP cohort: cyclophosphamide day 1, 40 mg/kg i.v.; doxorubicin day 1, 3.3 mg/kg i.v.; vincristine day 1, 0.5 mg/kg i.v.; prednisone from day 1 to day 5, 0.2 mg/kg per OS; etoposide day 1 and day 3, 3.3 mg/kg i.v., dasatinib from day 1 to day 5 and from day 8 to day 12, 30 mg/kg per OS.

The doses of cyclophosphamide, doxorubicin, vincristine and prednisone used were reported to be the maximum tolerated doses (MTD) in mice.^13,18–20^ The doses of etoposide and dasatinib used are lower than the MTD.^21–23^ The treatment scheme was designed to mimic the one used in patients.

OCI-Ly12-injected mice per cohort: ctrl *n* = 8, DA *n* = 8, CHOEP *n* = 7, DA + CHOEP *n* = 9. HD-MAR-2-injected mice per cohort: ctrl *n* = 8, DA *n* = 8, CHOEP *n* = 8, DA + CHOEP *n* = 8.

Cyclophosphamide, doxorubicin, vincristine and etoposide were diluted in saline and intravenously injected. Prednisone and dasatinib were diluted in water and administered per OS using an oral gavage. Tumour size and body weight were measured every 2 or 3 days. Tumour weights were calculated as g = (a × b^2^)/2, where a and b are the two largest perpendicular axes of the tumour nodule. At the end of the experiments, mice were euthanized by cervical dislocation. After sacrifice, tumour nodules were collected and formalin-fixed/paraffin-embedded (FFPE) for immunohistochemistry (IHC) analyses. Tumour growth inhibition (TGI) was defined as (1-(T/C)) × 100, where T and C represent the mean tumour weight in the treated and control groups, respectively. The study was conducted according to the Animal Research Reporting In Vivo Experiments (ARRIVE) requirements (Supplementary Methods).

### Immunohistochemistry

FFPE sections were stained with CD31 antibody and then incubated with a commercially available detection kit (EnVision™ FLEX + , Dako, Glostrup, Denmark) in an automated immunostainer (Dako Autostainer System), following the manufacturers’ instructions. Glass slides were then digitalised using the Aperio ScanScope XT (Leica Biosystems, Wetzlar, Germany) slide scanner. Image files were subjected to analyses, using the Aperio ImageScope software, version 11.1.2.760.

### Statistical analyses

All values were expressed as the mean ± standard deviation (SD), unless otherwise indicated. Graphs and statistical analyses were performed using GraphPad Prism 5 software (GraphPad Inc) and R software version 3.3.3. One-way ANOVA test was used for cell viability, Annexin V, TMRE assays and to assess significance of IHC staining. Tukey post hoc test was applied. Two-way ANOVA test was applied to cell cycle analyses and in vivo experiments. Comparisons were performed using Bonferroni’s multiple comparison test. The differences were considered significant at *P* < 0.05. Analyses of gene expression were performed as described.

## Results

### CHOEP treatment induces cell cycle perturbations and apoptosis in preclinical models of TCL

We first assessed the sensitivity of a panel of malignant T-cell lines (HH, cutaneous TCL; SUP-T1, lymphoblastic TCL; KARPAS-299, ALCL; JURKAT and HD-MAR-2, T-cell leukaemia) to CHOP, a combination of drugs reflecting the regimen used in patients.^13^ Escalating doses of CHOP were used, and viable cell counting performed by flow cytometry. CHOP significantly inhibited the proliferation of the cell lines in a dose-dependent and time-dependent manner with the best activity seen after 48 h of incubation and comparable half-maximal inhibitory concentration values (IC_50_) at this time point for all cell lines (not shown). Thereafter sensitivity to etoposide was determined: IC_50_ values were comparable for all cell lines (range 0.299 µM – 0.412 µM), with the exception of HH (0.089 µM) (not shown).

Thus, to test the efficacy of CHOEP combination, CHOP was combined with etoposide. Adding etoposide (used at 0.3 µM, corresponding to the mean IC_50_ for all cell lines) to CHOP (CHOEP 1 × ) strongly improves the anti-proliferative activity (Fig. S1A) when compared with either CHOP or etoposide treatment alone, although responses were heterogeneous among the cell lines (CHOEP IC_50_ values range 0.32 × −1.12 ×), with HH cells being the most sensitive and HD-MAR-2 the least sensitive (Table S1). Cyclophosphamide is known to be inactive in vitro as the production of the active metabolite is mediated by the cytochrome P450 in the liver.^24^ Accordingly, when the drug was titrated in all cell lines (range 100 pM–1 mM), its effects on cell proliferation were observed only at micromolar concentrations (100 µM for HD-MAR-2, JURKAT and SUP-T1; 250 µM for KARPAS-299; 500 µM for HH; data not shown). However, when we combined cyclophosphamide (C) with doxorubicin (H), vincristine (O) or prednisone (P), all combinations (CH, CO or CP) were slightly more active than the single agents in reducing the cell growth (p:ns), with the best efficacy achieved by combining all drugs (CH vs CHOP *p* < 0.05; CO vs CHOP p:ns; CP vs CHOP *p* < 0.01). The effect was further augmented by the addition of etoposide (CHOP vs CHOEP *p* < 0.001) (Fig. S1B).

**Fig. 1:**
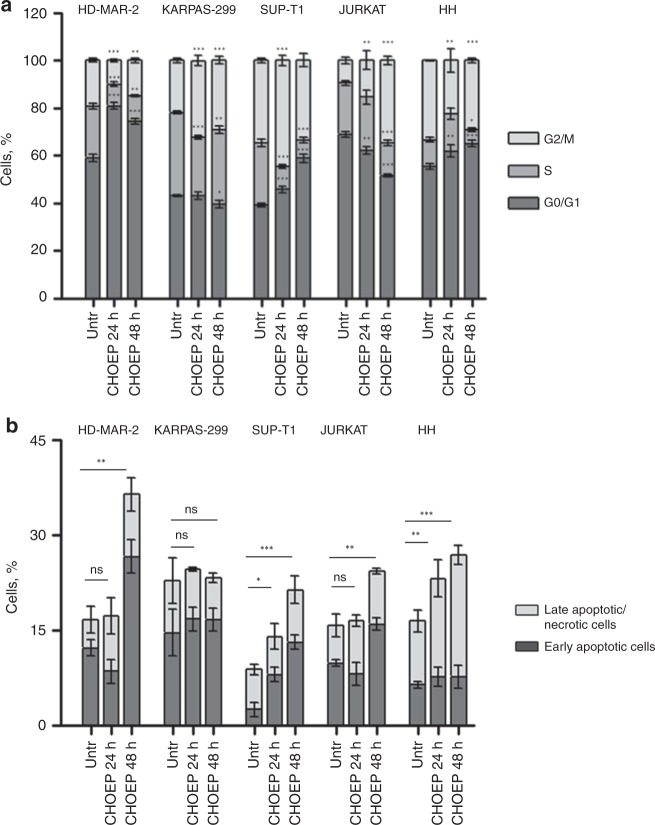
CHOEP treatment induces cell cycle perturbations and cell death in preclinical models of T-cell lymphoma. **a**, **b** Cell lines were exposed to IC_20_ CHOEP for 24 h and 48 h. Cell cycle distribution (**a**) and FITC–Annexin V/PI staining (**b**) were evaluated by flow cytometry. In panel **b**, early apoptotic cells are Annexin V positive/PI negative, late apoptotic/necrotic cells are Annexin V positive/PI positive. The data are the mean ± SD of three independent experiments. Asterisks indicate statistically significant differences (*p < 0.05; **p < 0.01; ***p < 0.001). In panel **a**, differences are referred to the untreated sample. In panel **b**, statistics was calculated on the total number of dead cells

Then, the effect of suboptimal concentrations of CHOEP on cell cycle progression was evaluated (Fig. 1a), and different modulations were observed across the cell lines. Twenty-four hours of exposure significantly increased the G0/G1 HD-MAR-2 cell cycle arrest (80.8 ± 1.4% vs 59 ± 1.6%, mean ± SD, *p* < 0.001) and decreased the S-phase in SUP-T1 (9.6 ± 0.7% vs 26 ± 1.7%, mean ± SD, *p* < 0.001) and KARPAS-299 cells (24.4 ± 0.8% vs 34.9 ± 0.6%, mean ± SD, *p* < 0.001). Exposure to CHOEP for up to 48 h gave more remarkable effects in all cell lines: G0/G1 cell cycle arrest was induced in HD-MAR-2, SUP-T1 and HH, at the expense of the S and G2/M phases (mean increase of G0/G1: 15, 19.7 and 9.6, respectively), while a decrease was observed in JURKAT cells (mean of 17.4%). As expected, CHOEP modulation of cell cycle was associated with increased apoptotic and necrotic cell death that was more significant at 48 h of CHOEP exposure in HD-MAR-2, SUP-T1, JURKAT and HH (early apoptotic cells: 15.9 ± 3.9% vs 7.8 ± 7.3%, mean ± SD, *p* < 0.01, and late apoptotic/necrotic cells: 11.4 ± 6.7% vs 5 ± 2.6%, mean ± SD, *p* < 0.01), but not in KARPAS-299 (Fig. 1b).

### CHOEP treatment modulates the signalling downstream of the TCR and increases the activity of tyrosine kinases in preclinical models of TCL

**Fig. 2:**
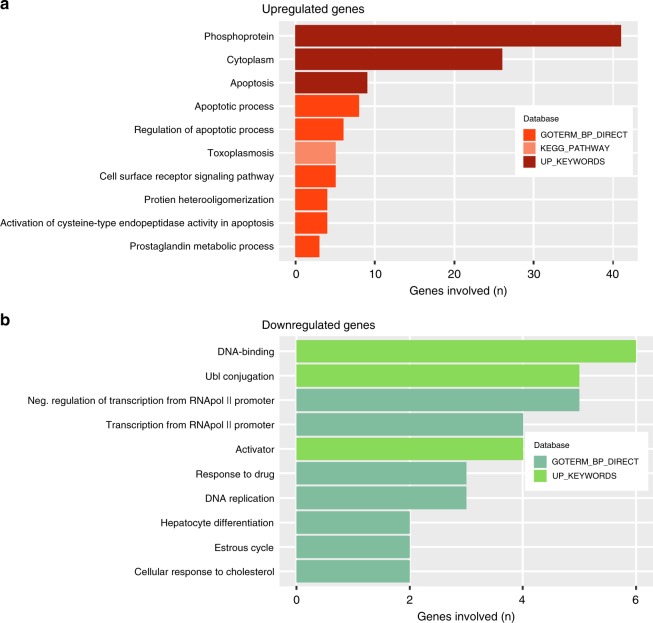
Gene-enrichment analysis upon CHOEP treatment in malignant T-cell lines. Top ten biological terms identified by the DAVID web tool significantly enriched in the 73 upregulated (**a**) and 17 downregulated (**b**) genes in treated cell lines (FC ≥2 - FDR < 0.05 and FC ≤ −2 - FDR < 0.05, respectively)

To gain insights into the molecular mechanisms underlying CHOEP effects and to unveil specific genes and pathways modified by CHOEP exposure, the gene expression profiles of untreated controls and CHOEP-treated cell lines were compared. After differential expression analysis, two lists of 73 significantly upregulated genes and 17 significantly downregulated genes were obtained (FC CHOEP vs untr ≤ −2 or ≥ 2 and FDR < 0.05, in at least two T-cell lines, Table S2). Pathway enrichment analysis performed with DAVID web tool showed that CHOEP significantly affects biological processes related to cell death, cell signalling, metabolic processes, regulation of transcription and DNA replication according to gene-enrichment analysis conducted using the DAVID web tool (Fig. 2a, b). This analysis highlighted that most of the upregulated genes (41/73) in treated cells, encode for phosphoproteins. These are mainly anti-proliferative and pro-apoptotic proteins involved in cell stress response, but also proteins triggering pro-survival signals, such as nuclear factor interleukin-3-regulated protein (*NFIL3*), protein kinase C zeta (*PRKCZ*), proline-rich protein 5 (*PRR5*) and GRB2-related adapter protein 2 (*GRAP2*). Of note, *GRAP2* encodes for a member of the Grb2 family of adaptor proteins that recruits signalling molecules leading to TCR activation.^25–27^ In addition, among genes of the “cell surface receptor signalling” pathway, *CD53* and *CD160* are upregulated by CHOEP treatment. *CD160* plays a role in regulating the activation of TCR proximal signalling interacting with the tyrosine kinase Lck and promoting the PI3K/AKT pathway.^28–30^

One likely explanation for the upregulation of these genes is that cells try to counteract the effects of CHOEP by activating phospho-kinase molecules that trigger survival signals mediated by the TCR. We therefore examined the modulation of the phosphorylation of 39 individual proteins involved in cellular proliferation and survival upon CHOEP treatment, using the Human Phospho-Kinase antibody array (R&D Systems). Representative phospho-array analysis of HD-MAR-2 treated cells (the least sensitive cell line to CHOEP), identified several pathways upregulated by CHOEP. These include the AKT/mTOR pathway (TOR and AKT kinases), MAPK signalling pathway (MSK1/2, MEK1/2, Erk1/2 and p38), SRC family kinase pathway (Lck and Fgr) and components of the β-catenin pathway (GSK α/β, FAK and β-catenin). In addition, several key transcription factors were upregulated, including STAT1, STAT3, STAT4, STAT5A/B, STAT6 and p53. Other signalling molecules that were upregulated include proteins of the DNA damage signalling pathway (Chk2 and p27) and the phospholipase C γ1 (PLCγ1), a key enzyme which links proximal and distal TCR signalling pathways^31^ (Fig. S2).

**Fig. 3:**
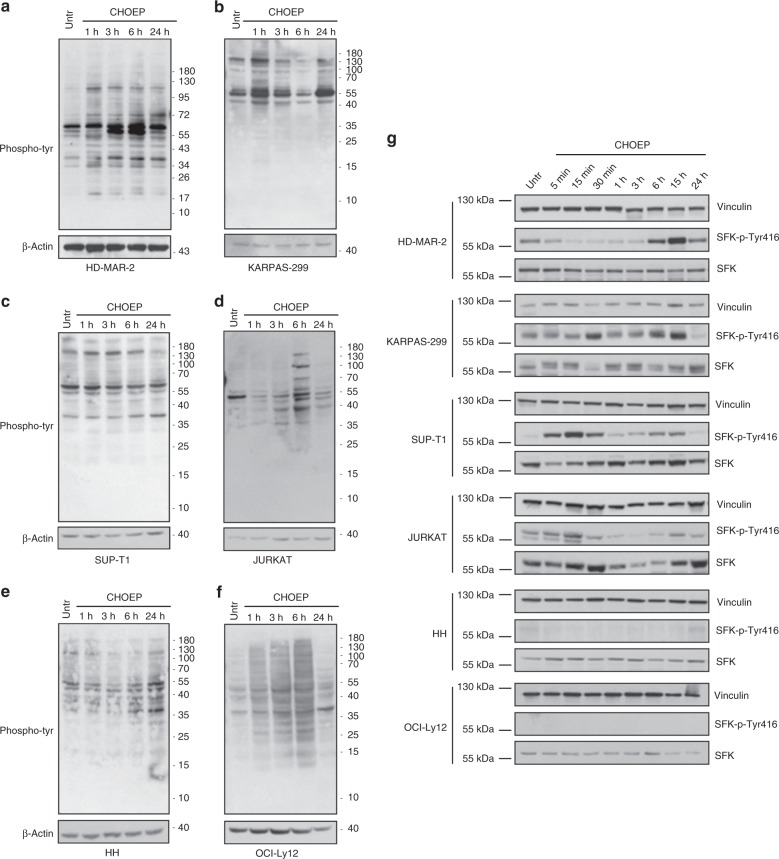
CHOEP treatment upregulates tyrosine phosphorylation levels and SRC family kinases activation. **a**–**f** Cell lines were treated with IC_20_ CHOEP for the indicated time points. Cell lysates were analysed by western blot with a pan phospho-tyrosine antibody and normalised on β-actin levels. **g** Cell lines were exposed to IC_20_ CHOEP for the indicated time points. Cell lysates were assayed by western blot for the activating phosphorylation of SRC family kinases (SFKs) on Tyr416. The data were normalised on total SFKs and vinculin levels

Collectively, these data suggest that survival signals from the TCR might contribute to determine chemotherapy sensitivity. To further investigate tyrosine kinase activity required for signal transduction through the TCR upon CHOEP treatment, western blot analysis using a pan phospho-tyrosine antibody was conducted. In all cell lines, CHOEP increased the level of tyrosine phosphorylation with different kinetics (Fig. 3a–e). This was also true for the OCI-Ly12 cell line that was included in the analysis as it represents one of the fewest available cell line derived from PTCL-NOS.^32^ CHOEP significantly inhibited the proliferation of OCI-Ly12 (IC_20_: 0.23×, IC_30_: 0.32×, IC_50_: 0.48×) and induced tyrosine phosphorylation as detected by western blot analysis of treated cells (Fig. 3f). Of note, all western blot analysis revealed an increase in the phosphorylation signals of 50–60 kDa proteins, suggesting that CHOEP might induce the TCR signalling through SRC-family kinases activation (SFKs) (Fig. 3a–f). The modulation of SFKs phosphorylation was then evaluated by western blot. SFKs are activated upon CHOEP treatment in HD-MAR-2, KARPAS-299, SUP-T1 and JURKAT cells, although each cell line displayed a specific kinetics of activation (Fig. 3g). No SFK phosphorylation was detected in HH and OCI-Ly12 cell lines, which, of note, are those cell lines displaying the highest sensitivity to CHOEP (Fig. 3g). Nonetheless, when the activation of additional signalling molecules downstream the TCR such as ZAP-70, p38, MEK1/2, ERK1/2 and AKT was evaluated in all cell lines, in HH and OCI-Ly12 we observed the modulation of both ERK1/2 and AKT phosphorylation (Fig. S3A) upon treatment with CHOEP. No other significant modulation was observed, with the exception of an increase in AKT phosphorylation in SUP-T1 cell line after 3, 6 and 24 h of CHOEP exposure (data not shown). Notably, when we analysed the total tyrosine kinase activity and SFKs activating phosphorylation in CHOEP-resistant JURKAT cells, we observed that, compared with the parental cell line, chemoresistant cells were characterised by a significant increase in basal phosphorylation levels, which were not further augmented upon CHOEP administration (Fig. S3B). These results indicate that even chronic exposure to CHOEP activates signalling kinases belonging to TCR signalling. In line with this, the engagement of the TCR in JURKAT cells by phytohaemagglutinin (PHA, 1 µg/ml) that resulted in cell rosetting, upregulation of CD69 expression and upregulation of pan tyrosine phosphorylation (data not shown), seems to contribute to chemoresistance. In fact, PHA-stimulated cells exposed to IC_50_ CHOEP were less sensitive to CHOEP than unstimulated cells exposed to the same concentration of CHOEP (growth inhibition of PHA-stimulated cells 11 ± 8.7% mean ± SD, p:ns, vs 36.1 ± 5.8% mean ± SD of unstimulated cells, *p* < 0.001; data are expressed as percentages of untreated cells; data not shown).

### The tyrosine kinase inhibitor dasatinib potentiates CHOEP effects in malignant T-cell lines

**Fig. 4:**
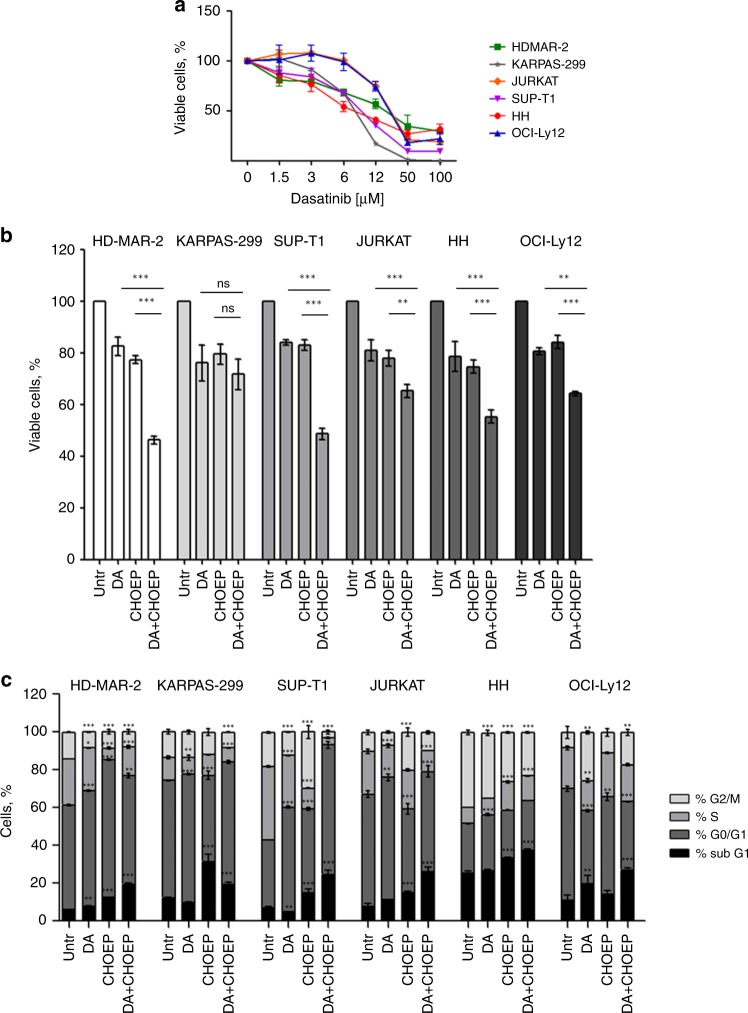
Dasatinib addition to CHOEP potentiates the anti-proliferative effect of chemotherapy. **a** Cell lines were incubated with the indicated doses of dasatinib. After 48 h, cell viability was monitored by flow cytometry. **b**, **c** Cells lines were treated with IC_20_ CHOEP and IC_20_ dasatinib (DA) either alone or in combination for 48 h. The percentage of viable cells (**b**) and cell cycle distribution (**c**) were assessed by flow cytometry. The data represented are the mean ± SD of at least two independent experiments. In **a** and **b**, the data are expressed as a percentage of untreated samples. Asterisks indicate statistically significant differences (*p < 0.05; **p < 0.01; ***p < 0.001; ns: not significant). In **c**, differences are referred to the untreated sample

As all the above reported findings prompt to a role of the TCR signalling pathway activation in promoting pro-survival signals mainly mediated by SFKs in response to chemotherapy in TCL preclinical models, we hypothesise that the addition of a tyrosine kinase inhibitor to CHOEP might improve response rates of TCLs. To test this hypothesis, we first exposed cells to increasing concentrations of the TKI dasatinib (1.5–100 µM) for 48 h. A significant dose-dependent decrease in cell proliferation was detected in all cell lines (Fig. 4a), and dasatinib inhibitory concentrations (ICs) were then calculated (Table S3). As expected, upon dasatinib exposure, SFKs phosphorylation was totally abrogated (as shown in representative Fig. S4A), confirming that these kinases are well targeted by the inhibitor.^33^

To define the effects of dasatinib combined with CHOEP at the cellular level, we assessed the cell proliferation and cell death in cell lines treated with the dasatinib–CHOEP (DA + CHOEP) combination. As shown in Fig. 4b, the addition of DA to CHOEP significantly inhibited cell proliferation in HD-MAR-2, SUP-T1, JURKAT, HH and OCI-Ly12 cells (mean inhibition: DA 18.6 ± 1.5%, range 16–21.3%; CHOEP 20.6 ± 4% range 15.8–25.3%; DA + CHOEP 44 ± 8.7% range 34.7–53.7%; mean ± SD, *p* < 0.001), but not in KARPAS-299 cell line (DA 23.9 ± 6.9%; CHOEP 20.4 ± 3.8%; DA + CHOEP 28.2 ± 5.9%, mean ± SD, p:ns) compared with the exposure to single agents.

Cell cycle analysis (Fig. 4c) indicated that the DA + CHOEP combination induced G1-phase cell cycle arrest and accumulation of a sub-G1-phase population in all cell lines, indicative of the presence of dead cells characterised by fragmented DNA (mean fold DA/untr 1.8 ± 4, CHOEP/untr 8.7 ± 5.5, DA+CHOEP/untr 14.1 ± 4.2). Of note, CHOEP and dasatinib, used either alone or combined, did not affect cell growth of normal T lymphocytes isolated from the peripheral blood of healthy donors (Fig. S4B).

The activity of two other TKIs, sorafenib and ruxolitinib, were additionally tested in combination with CHOEP. Sorafenib is an oral multi-kinase inhibitor approved for patients with advanced renal cell carcinoma and hepatocellular carcinoma^34^ with potent activity against several receptor tyrosine kinases involved in tumour progression and tumour angiogenesis.^35^ Ruxolitinib is instead a selective JAK inhibitor recently approved for the treatment of primary and secondary myelofibrosis.^36^ Cell lines were exposed to escalating concentrations of sorafenib and ruxolitinib for 48 h, and relative ICs were obtained (Table S4). We then investigated the anti-proliferative activity of each inhibitor in combination with CHOEP. As shown in Fig. S5A, sorafenib, similarly to dasatinib, significantly reduced cell proliferation in all cell lines when used in combination with CHOEP (mean inhibition: SO 16.3 ± 1.7%, range 14.3–19.4%; CHOEP 17.8 ± 5.4% range 9.4–24.2%; SO+CHOEP 45.1 ± 7.5% range 32.3–47.8%; mean ± SD, *p* < 0.001). On the other hand, the addition of ruxolitinib (RU) to CHOEP did not significantly reduce cell proliferation in malignant T-cell lines with the exception of JURKAT cells (CHOEP 26 ± 5.7%; RU+CHOEP 60 ± 2.8%; mean ± SD, *p* < 0.01) (Fig. S5B) that are in fact characterised by the upregulation of the JAK/STAT pathway.^37^

### Dasatinib enhances CHOEP-induced apoptotic cell death in preclinical models of T-cell lymphoma

**Fig. 5:**
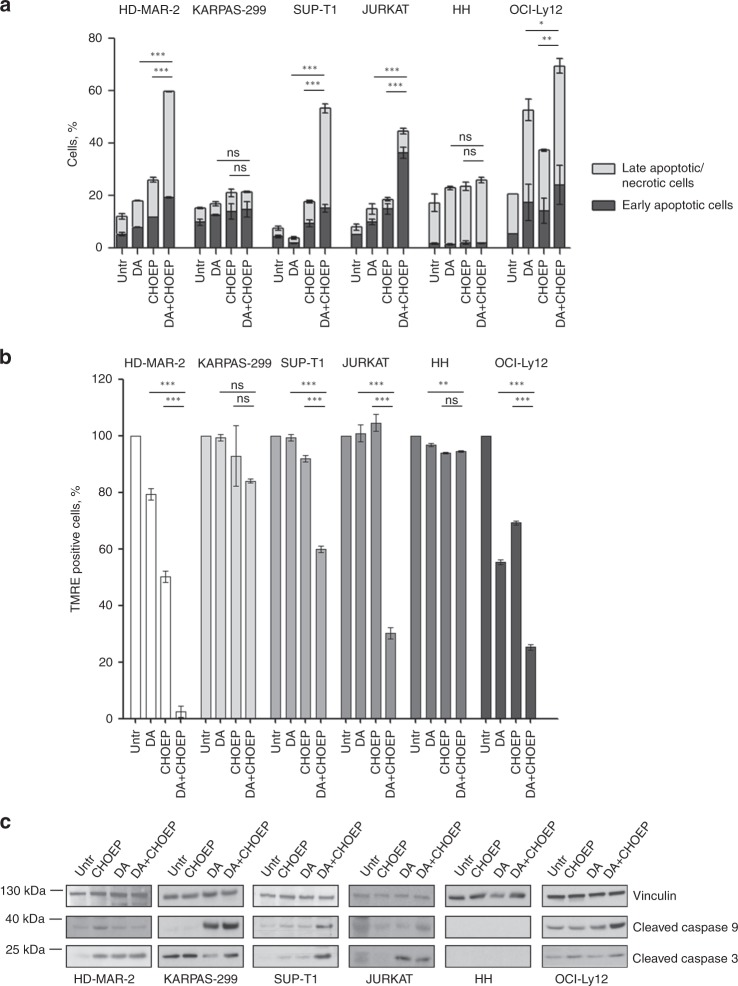
Dasatinib addition to CHOEP induces apoptotic cell death. **a**–**c** Cells lines were treated with IC_20_ CHOEP or IC_20_ dasatinib (DA) alone or in combination for 48 h. Then cells were stained with the FITC–Annexin V/PI (**a**) or the fluorescent probe TMRE (**b**) and monitored by flow cytometry to detect apoptotic cell death and the mitochondrial membrane depolarisation, respectively. In **a**, early apoptotic cells are Annexin V positive/PI negative, late apoptotic/necrotic cells are Annexin V positive/PI positive. Statistics is calculated on the total number of dead cells (early+late apoptotic cells). In **b**, the data are expressed as a percentage of untreated samples. The data represented are the mean ± SD of at least two independent experiments. Asterisks indicate statistically significant differences (*p < 0.05; **p < 0.01; ***p < 0.001: ns: not significant). **c** Upon treatments, cell lysates were prepared and assayed by western blot using the indicated antibodies

The mechanism of DA+CHOEP-induced cell death was further investigated in all cell lines. Compared with single treatments, the combination caused increased cell death (median increase of early + late apoptotic cell death: DA 8.2 ± 12.6%; CHOEP 11.5 ± 3.4%; DA+CHOEP 44.2 ± 5.6%; *p* < 0.001) of HD-MAR-2, SUP-T1, JURKAT and OCI-Ly12 cell but not of KARPAS-299 and HH, as detected by staining cells with FITC–Annexin V and propidium iodide (PI) and flow cytometry analysis (Fig. 5a). The increase in cell death was also associated with a severe mitochondrial depolarisation. As shown in Fig. 5b, the percentage of cells positive to TMRE (viable cells, not subjected to a depolarisation of the mitochondrial membrane, usually linked to apoptosis induction) was significantly reduced in HD-MAR-2, SUP-T1, JURKAT and OCI-Ly12 cells treated with the DA+CHOEP combination (mean percentage of TMRE-positive cells: DA 83.2 ± 20.8%, CHOEP 78.5 ± 23.2%, DA+CHOEP 29.2 ± 24%; mean ± SD, *p* < 0.001) but not in KARPAS-299 and HH. Interestingly, treatment with either DA or CHOEP alone reduced the percentage of TMRE-positive cells only in HD-MAR-2 and OCI-Ly12 cell lines. In addition, in KARPAS-299 and JURKAT cell lines dasatinib used either alone or in combination, induced caspase activation (caspase 9 and 3, respectively), whereas in SUP-T1 and OCI-Ly12 only the combination DA+CHOEP-induced caspase 9 and 3, respectively. In HH cell line, no caspase activation was detected when CHOEP and dasatinib were either used alone or combined (Fig. 5c).

To rule out the possibility that dasatinib activity could be related to the presence of activating mutations in genes related to TCR signalling such as *FYN* and *RHOA* that have been recently reported in a significant fraction of PTCL patients,^38^ Sanger sequencing was performed. When we checked for the presence of the L174R, R176C and Y531H mutation in the *FYN* gene (Fig. S6A, S6B) and for the G17V mutation of *RHOA* (Fig. S6C), no substitution was detected in the cell lines.

### DA+CHOEP treatment suppresses tumour growth of TCLs in mice

**Fig. 6:**
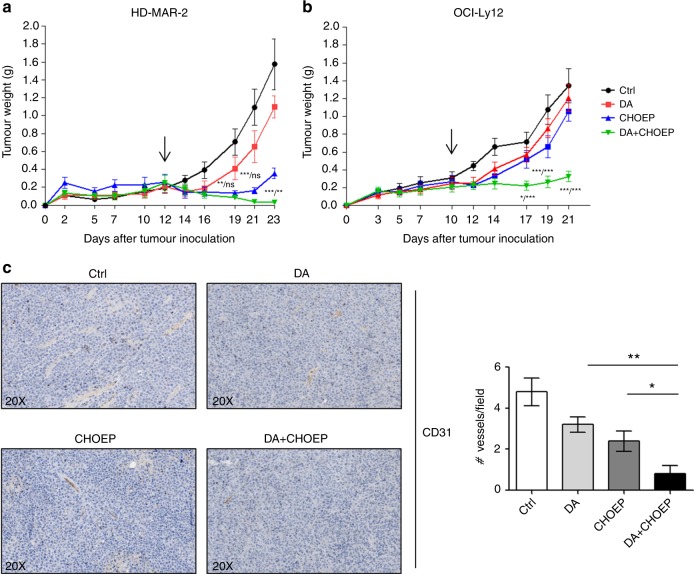
Anti-tumour activity of DA+CHOEP combination in xenograft models of T-cell lymphoma. **a**, **b** NOD/SCID mice were subcutaneously inoculated with HD-MAR-2 (**a**) or OCI-Ly12 cells (**b**). When tumour nodules reached 150–200 mm^3^, they were randomised to receive treatment with DA, CHOEP, DA+CHOEP or vehicle (ctrl) as described in the Methods section. The beginning of the treatment is indicated by the black arrow. The data are expressed as the mean ± SEM. Asterisks indicate statistically significant differences (*p < 0.05; **p < 0.01; ***p < 0.001: ns: not significant). For each time point, the first asterisk refers to the DA-DA+CHOEP comparison, the second one to CHOEP-DA+CHOEP. **c** Representative images of CD-31 immunostaining of OCI-Ly12 tumours excised at day 21. Left panel, original magnification 40×. Right panel, quantification of blood vessel number/field, calculated of ten randomly selected fields of each section

The in vivo anti-tumour activity of the combination DA+CHOEP was investigated in two localised human tumour xenografts models in NOD/SCID mice developed using subcutaneous injection of HD-MAR-2 (the least sensitive cell line) and OCI-Ly12, the PTCL cell line, which in in vitro experiments was among the most sensitive to CHOEP. The in vivo growth of HD-MAR-2 tumour nodules was significantly affected by CHOEP and by the combined DA+CHOEP treatment (ctrl 1.57 ± 0.28 g, CHOEP 0.36 ± 0.06 g, DA+CHOEP 0.03 ± 0.01 g; mean ± SEM, *p* < 0.001) but not by dasatinib used alone (ctrl 1.57 ± 0.28 g, DA 1.1 ± 0.12 g; mean ± SEM, p:ns) (Fig. 6a). The relative tumour growth inhibition was of 30.2% (DA), 77.4% (CHOEP) and 98.1% (DA+CHOEP). In contrast, in vivo treatment with either dasatinib or CHOEP slightly affected the growth of OCI-Ly12 nodules compared with untreated controls (DA 1.21 ± 0.15 g, CHOEP 1.06 ± 0.12 g, ctrl 1.34 ± 0.19 g; mean ± SEM, p:ns) (Fig. 6b). Despite the partial lack of activity of the single treatments, the combination significantly reduced tumour growth as compared with the untreated controls (DA+CHOEP 0.33 ± 0.06 g, ctrl 1.34 ± 0.19 g; mean ± SEM, *p* < 0.001), the use of dasatinib alone (DA+CHOEP 0.33 ± 0.06 g, DA 1.21 ± 0.15 g; mean ± SEM, *p* < 0.001) and the use of CHOEP (DA+CHOEP 0.33 ± 0.06 g; CHOEP 1.06 ± 0.11 g, mean ± SEM, *p* < 0.001), with tumour growth inhibition values of 10.2% (DA), 21.3% (CHOEP) and 75.7% (DA+CHOEP).

In addition, immunohistochemical detection of CD31 in the tumours generated by OCI-Ly12 injection showed a significantly low microvessel density in the DA+CHOEP-treated group, as compared with the other cohorts of mice (untr 4.8 ± 0.68, DA 3.2 ± 0.39, CHOEP 2.8 ± 0.44, DA+CHOEP 0.8 ± 0.42; mean ± SEM of the number of vessels/field) (Fig. 6c).

## Discussion

In an attempt to identify possible pro-survival mechanisms activated by TCLs to counteract the cell death pathways triggered by chemotherapy, we analysed the expression levels of genes induced by CHOEP treatment in a panel of TCL cell lines. Among others, CHOEP induced the upregulation of genes encoding for pro-survival proteins and for signalling molecules downstream of the TCR that was also associated with an increase in protein tyrosine phosphorylation. These results are in line with the idea that malignant T cells use growth and survival signals provided by the TCR.

Consistent with this, several lines of evidence suggest that TCR activation might be as relevant to TCL pathogenesis as the constitutive B-cell receptor (BCR) signalling is known to promote neoplastic B-cell survival and expansion in B-cell lymphomas. This view is supported by the widespread expression of the TCR and of its associated kinases in different subtypes of PTCLs^39^ and by the presence of gene fusions, translocations, point mutations, small indels, as well as amplifications and deletions that lead to the constitutive activation of the TCR.^40–44^ In addition, the mutational status of TCR-related genes and the engagement of TCR on tumour T cells seem to have clinical implications as they have been identified as distinguishing features of poor prognosis subgroups of PTCLs.^45,46^ Thus, pharmacologic inhibition of the TCR and/or of its associated kinases using multi-tyrosine kinase inhibitors (TKIs) might represent a strategy to augment response rates and decrease chemotherapy resistance.

Dasatinib, an orally administered well-tolerated small molecule inhibitor of multiple tyrosine kinases^11^ used for the treatment of chronic myeloid leukaemia (CML) and acute lymphoblastic leukaemia (ALL) has been tested as a single agent in a phase I/II study for relapsed/refractory NHL.^12^ Of note, the only complete responses were observed in two PTCL-NOS patients.^47^ Despite these encouraging results, dasatinib treatment for PTCL has not moved forward. This might be due to the fact that monotherapy with selective targeted agents is unlikely to prove fully effective when used alone and most anti-tumoural therapies are based on combinations.

Here, we provide preclinical evidence that the anti-tumour efficacy of the combination of dasatinib and the anthracycline-based regimen CHOEP is augmented when compared with either chemotherapy or dasatinib as monotherapy. The effect is mediated by the induction of cell cycle perturbations, mitochondrial injury and caspase-dependent apoptosis in malignant T-cell lines. These findings were also confirmed in animal models where no toxic effects were observed. Of interest, the addition of dasatinib to CHOEP improves efficacy in any T-cell lymphoma and leukaemia model used irrespective of: (i) the biological differences among histologies; (ii) the response to CHOEP administered alone; (iii) the presence of activating mutations in genes of the TCR signalling pathway. This broad activity demonstrated by the DA+CHOEP combination can be explained by the fact that dasatinib targets many of the signalling pathways subverted in T-cell oncogenesis such as ABL, the SRC family kinases (SFKs), PDGFR alpha and beta.^33^ SRC inhibition affects TCR ability to interact with the PIK3/AKT, RAS/MAPK and STAT3 pathways, and to modulate cell migration and invasion through interaction with integrins, the focal adhesion kinase (FAK), and regulators of the family of Rho-GTPases.^44,48,49^ Accordingly, sorafenib another oral inhibitor of several receptor tyrosine kinases involved in tumour progression and tumour angiogenesis approved for the treatment of advanced renal cell carcinoma and hepatocellular carcinoma,^50^ exerts similar effects as dasatinib on malignant T cells, whereas ruxolitinib a selective inhibitor of JAK1 and JAK2 does not. Collectively data presented clearly indicate that dasatinib warrants testing in combination with anthracycline-based programs for the upfront treatment of TCLs to improve outcome.

## Supplementary information

Supplementary Files

## Data Availability

The data generated during the current study are available from the corresponding author upon reasonable request. Gene expression data has been deposited in the NCBI Gene Expression Omnibus database, accession number GSE129289.
