# Supplementary material for: Tyrosine kinase inhibition to improve anthracycline-based chemotherapy efficacy in T-cell lymphoma
Source: Br J Cancer. 2019 Sep 2;121(7):567–77. doi: 10.1038/s41416-019-0557-8 (PMC6889385; doi:10.1038/s41416-019-0557-8)
Supplement: Supplementary file 1 — Supplementary Files [file 41416_2019_557_MOESM1_ESM.pdf]

A

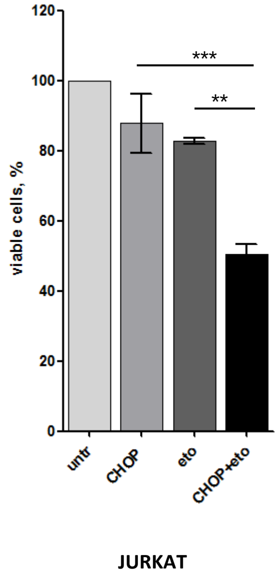

B

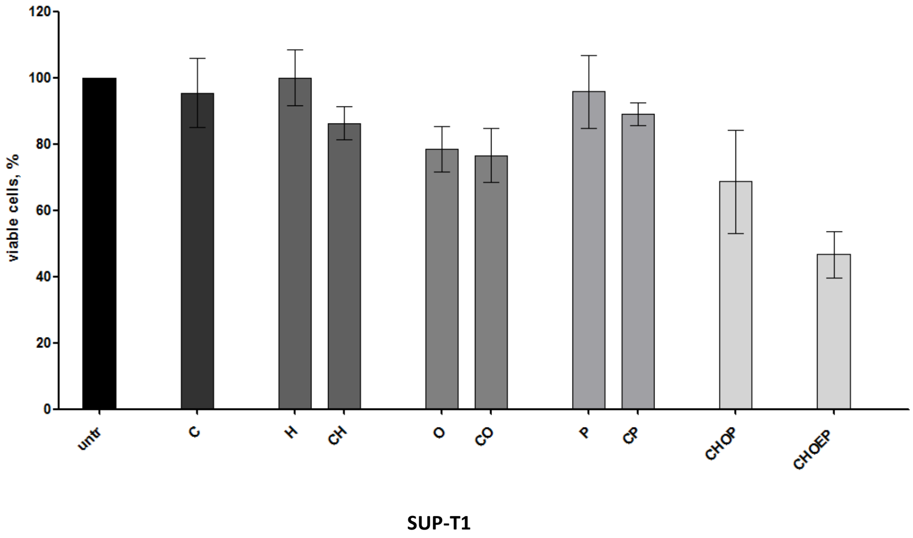

Figure S1. Effects of CHOEP on cell viability in preclinical models of T-cell lymphoma.
(**A**) Representative experiment performed in JURKAT cells exposed to IC_{20} CHOP, IC_{20} etoposide (eto), or IC_{20} CHOP+IC_{20} eto. (**B**) Representative experiment performed in SUP-T1 cells treated with each of the components of the CHOP regimen used alone (C, H, O and P) or in combination (CH, CO, CP; CHOP and CHOEP). Doses were the ones of IC_{50} CHOEP. In **A** and **B**, 48h upon treatments viable cell counting was performed by flow cytometry. Data are expressed as a percentage of untreated samples and are the mean±SD of at least two independent experiments. Asterisks indicate statistically significant differences ** $p<0.01$; *** $p<0.001$).

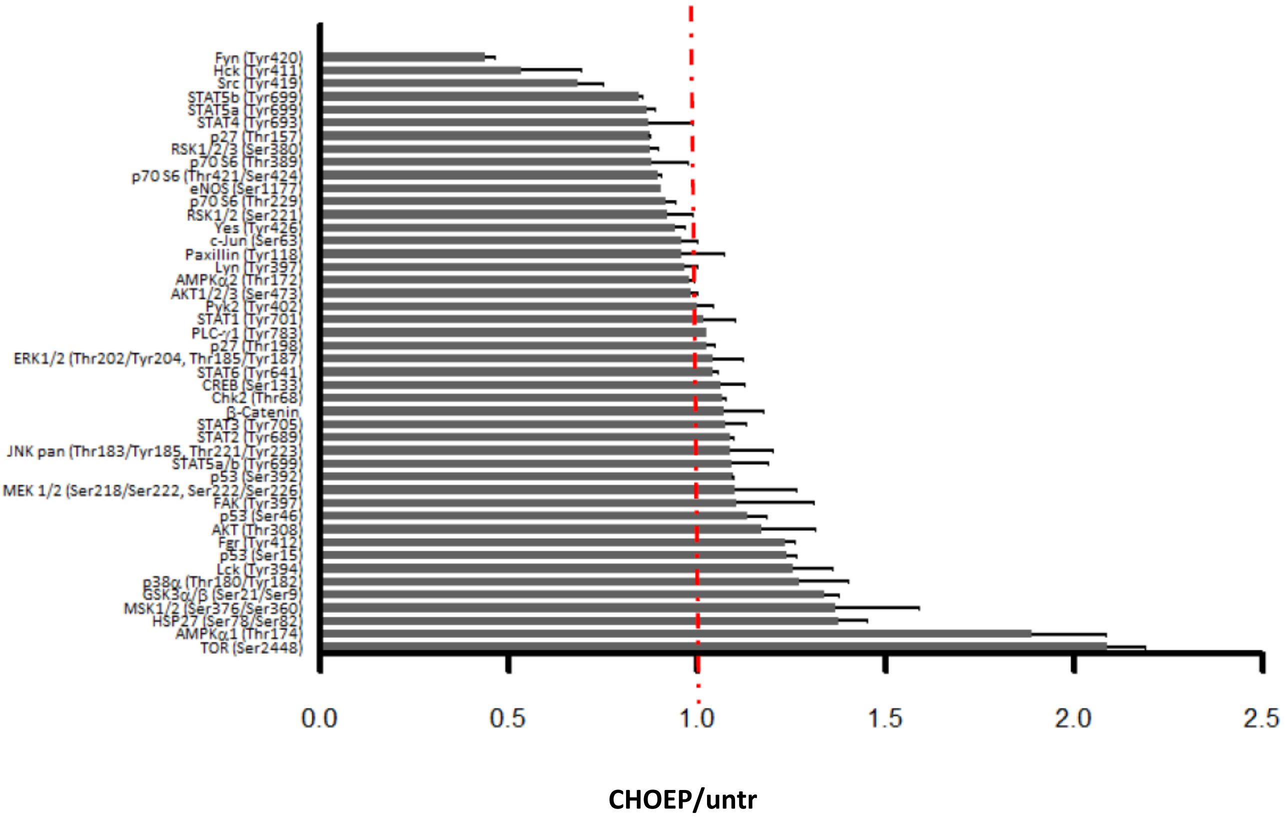

Figure S2. Results of the Proteome Profiler Human Phospho-Kinase Array in HD-MAR-2 cell line.

Representative Proteome Profiler Human Phospho-Kinase Array performed using cell lysates of HD-MAR-2 cells, treated or not with IC_{20} CHOEP for 3h. The graph shows the results of the densitometric analysis of spots obtained, normalized as described in methods.

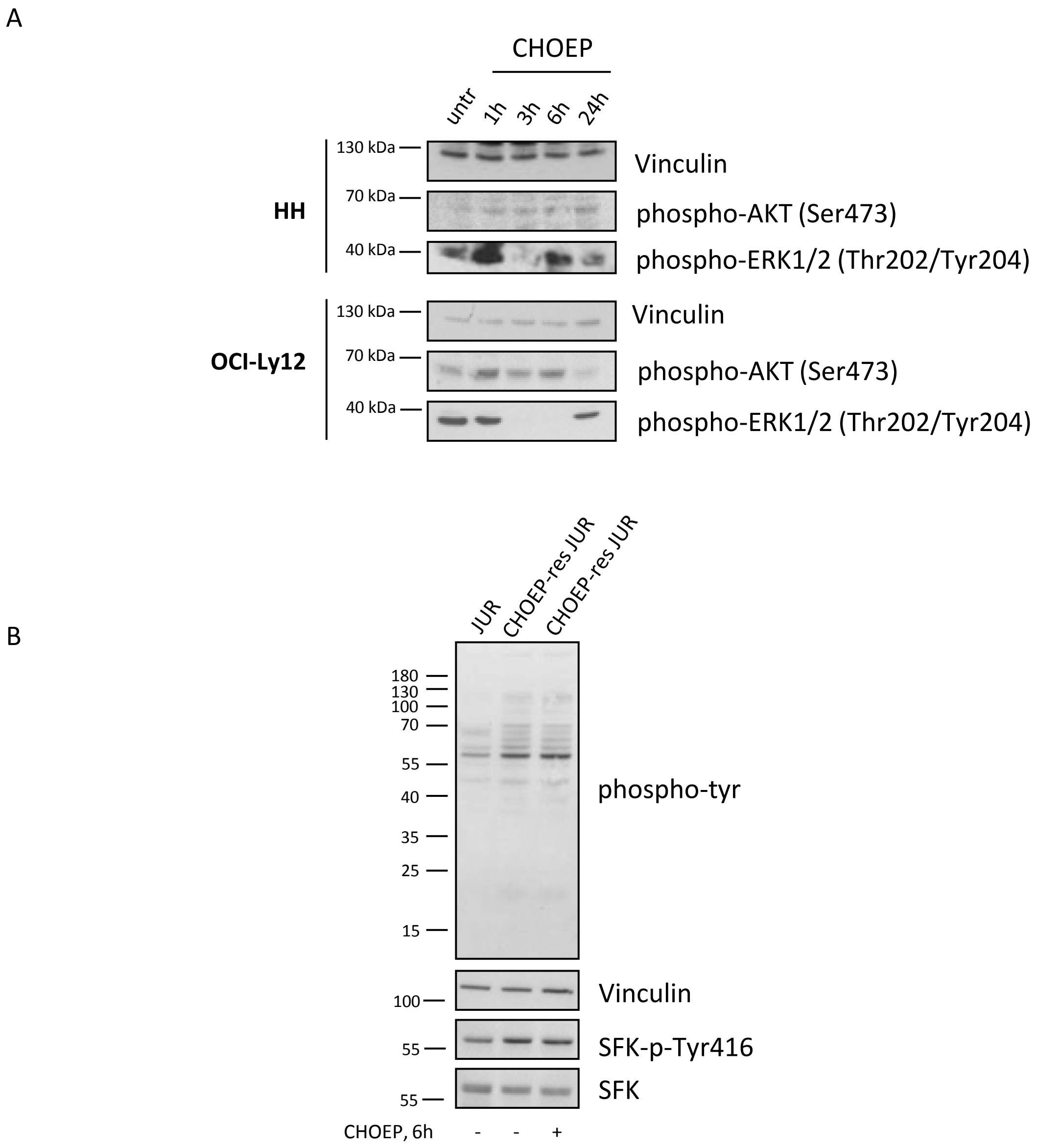

Figure S3. Modulation of TCR downstream molecules in malignant T-cell lines upon CHOEP exposure.

(**A**) HH and OCI-Ly12 cell lines were treated with IC_{20} CHOEP for the indicated time points and cells lysates were analysed by western blot with phospho-AKT and phospho-ERK1/2 antibodies and normalized on Vinculin levels. (**B**) Western blot showing pan tyrosine phosphorylation and SFKs phosphorylation on Tyr416 in JURKAT and CHOEP-resistant JURKAT cells, treated with IC_{50} CHOEP for 6 hours.

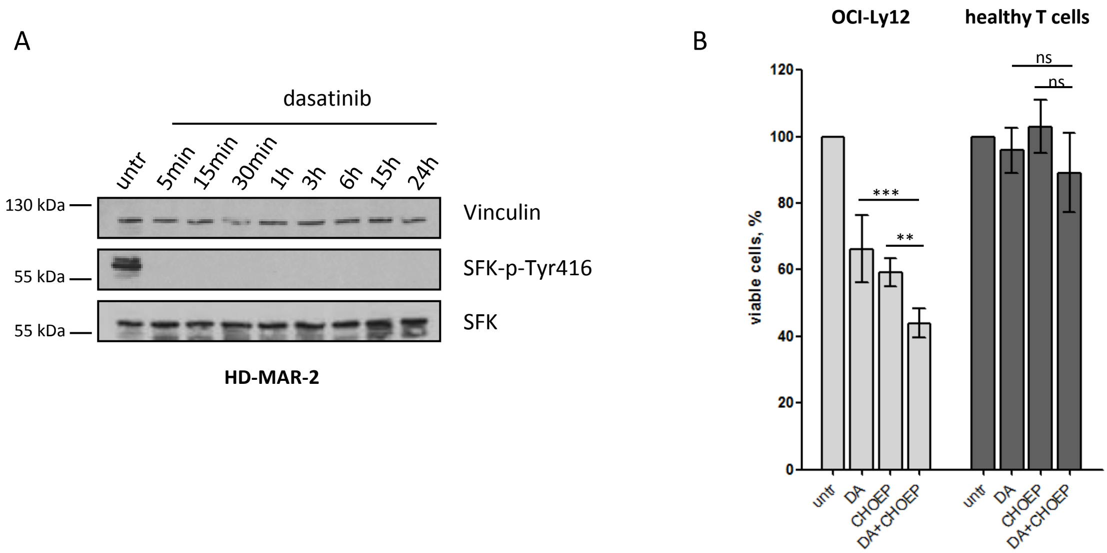

Figure S4. Effects of dasatinib alone and in combination with CHOEP.

(**A**) Representative western blot of HD-MAR-2 cells treated with IC_{20} dasatinib for the indicated time points. (**B**) OCI-Ly12 and healthy T lymphocites (CD3+, isolated from the peripheral blood of healthy donors) were treated with IC_{50} CHOEP and IC50 dasatinib alone or in combination. After 48h viable cells were counted by flow cytometry. Data are expressed as a percentage of untreated samples and are the mean±SD of three independent experiments. Asterisks indicate statistically significant differences (** p<0.01; *** p<0.001; ns: not significant).

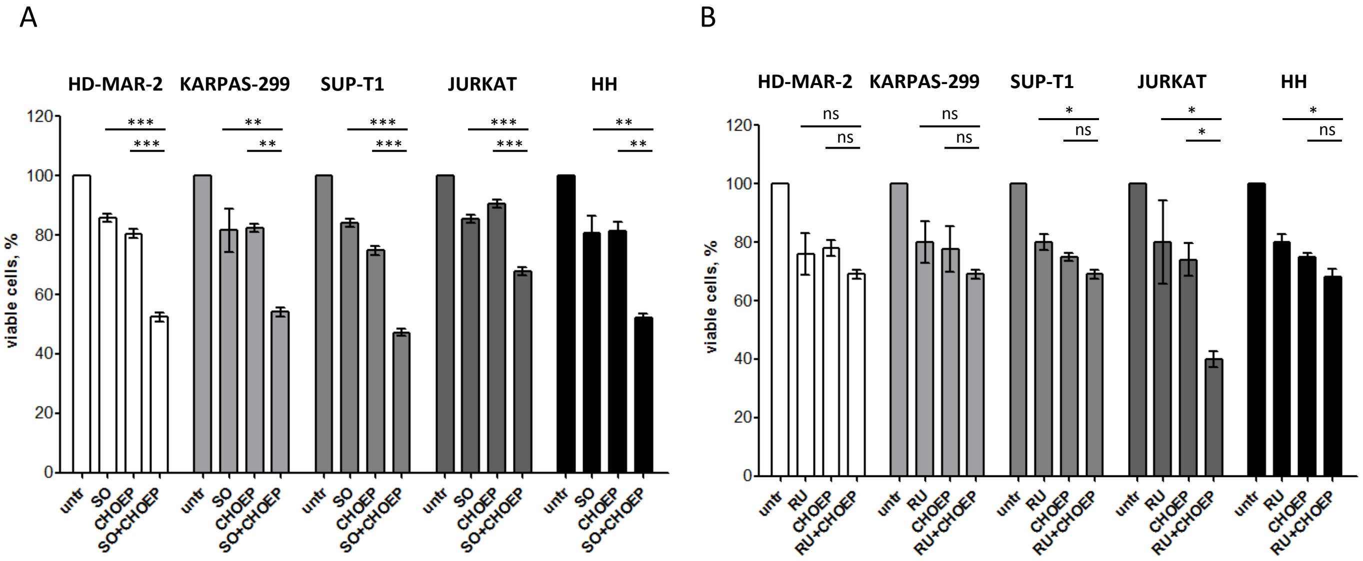

Figure S5. Effects of sorafenib and ruxolitinib, alone and in combination with CHOEP, on the viability of malignant T-cell lines.

Cells were exposed to IC_{20} sorafenib (SO), IC_{20} CHOEP or IC_{20} SO+IC_{20} CHOEP in (**A**) and to IC_{20} ruxolitinib (RU), IC_{20} CHOEP or IC_{20} RU+IC_{20} CHOEP in (**B**). After 48h viable cells were counted by flow cytometry. Data are expressed as a percentage of untreated samples and are the mean±SD of two independent experiments. Asterisks indicate statistically significant differences (* $p<0.05$; ** $p<0.01$; *** $p<0.001$; ns: not significant).

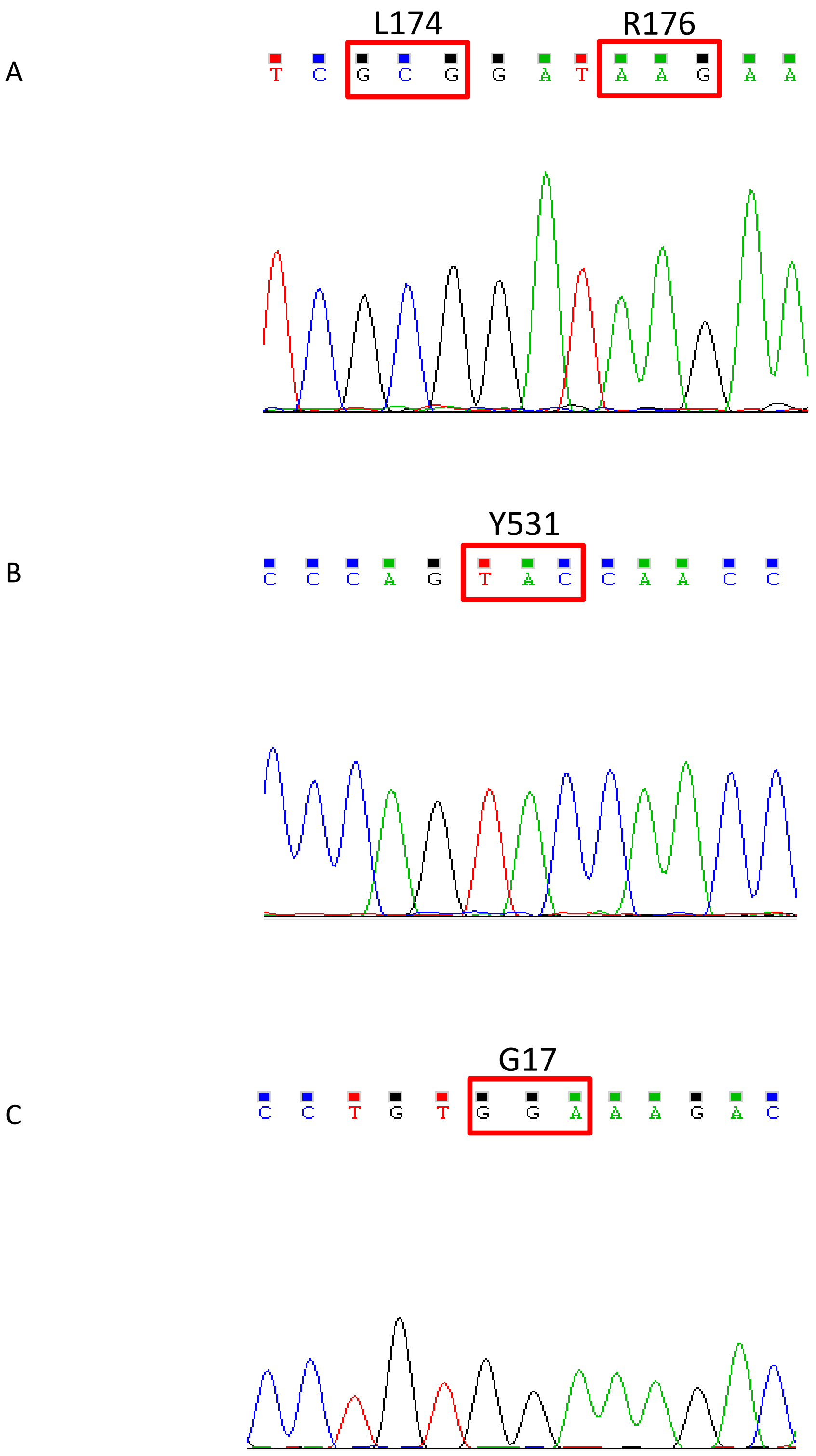

Figure S6. Dideoxynucleotide sequence analysis of *FYN* and *RHOA* mutations.
DNA sequencing chromatograms of OCI-Ly12 genomic DNA samples representative of the wild type allele sequences of *FYN* (A , B) and *RHOA* (C).

CHOEP			
**Cell line**	**IC_{20}**	**IC_{30}**	**IC_{50}**
HH	0.03X	0.08X	0.32X
JURKAT	0.32X	0.4X	0.56X
SUP-T1	0.4X	0.56X	0.81X
KARPAS-299	0.48X	0.64X	0.81X
HD-MAR-2	0.72X	0.88X	1.12X

Table S1. ICs values of CHOEP in malignant T-cell lines.
Cell lines were exposed to increasing concentrations of CHOEP for 48 hours. Cell viability was assessed by flow cytometry and ICs values were calculated with GraphPad Prism 5 software.

Table S2

up-regulated genes		down-regulated genes
TP53INP1	MXD4	PRODH
NFIL3	KLHL24	EGR1
GSTM1	SMAP2	TRIP13
RCAN1	CD160	E2F7
RGS1	DDIT4	RRM2
RHOBTB3	BTG1	CDC25A
GPR56	TSC22D3	LRP8
KLF2	AKR1C2	DSCC1
TG	VAMP5	NR2C2AP
CRADD	TBCEL	MZT1
TOX2	PMAIP1	ANXA1
HMHB1	SNX30	CA2
TMEM187	KCNK1	NFE2L3
BIK	LAMA3	IL22
CROCC	TUBA3C	BHLHE40
PFKFB2	TUBA3E	EPAS1
ABCC5	ZADH2	HMGCS1
LINC00324	KLF9	
TRANK1	IL7R	
HIST1H2BD	AKR1C3	
GATSL3	NINJ2	
ITGA6	ERAP2	
UBASH3B	GLCCI1	
HIST2H2BE	CASP8	
KDM5B	ISG20	
TRAF3IP2	FKBP5	
P2RX1		
PRKCZ		
BTG2		
TBL1X		
RPS29		
RPL13A		
YPEL5		
GRAP2		
EOMES		
PRR5		
RCSD1		
BIRC3		
GLTSCR2		
MAL		
CELF2		
PIK3IP1		
BMF		
ZFP36L2		
FBXO32		
CD53		
SOCS1		

dasatinib (µM)			
**Cell line**	**IC_{20}**	**IC_{30}**	**IC_{50}**
HD-MAR-2	1.4	2.6	6.5
HH	1.6	2.4	4.6
SUP-T1	3.6	4.8	7.3
KARPAS-299	5.2	6	7.7
JURKAT	8.4	10.4	14.7
OCI-Ly12	8.6	10.7	15

Table S3. ICs values of dasatinib in malignant T-cell lines.
Cell lines were exposed to increasing concentrations of dasatinib for 48 hours. Cell viability was assessed by flow cytometry and ICs values were calculated with GraphPad Prism 5 software.

Cell line	IC_{20}	IC_{30}	IC_{50}
**sorafenib (μM)**			
KARPAS-299	5.5	6.1	7.1
JURKAT	5.7	7.3	10.6
SUP-T1	7.7	8.6	10
HD-MAR-2	8.1	9.5	12
HH	8.8	11.6	18
**ruxolitinib (μM)**			
HH	1	2	6
KARPAS-299	2.3	3.1	5
SUP-T1	3.5	4.8	8
JURKAT	3.6	5.1	8.8
HD-MAR-2	7.8	8.2	10.3

Table S4. ICs values of sorafenib and ruxolitinib in malignant T-cell lines.
Cell lines were exposed to increasing concentrations of sorafenib or ruxolitinib for 48 hours. Cell viability was assessed by flow cytometry and ICs values were calculated with GraphPad Prism 5 software.

Supplementary Methods: Animal studies in compliance with the ARRIVE guidelines

Ethical statement	Procedures involving animals and their care were conducted in conformity with national and international laws and policies (European Economic Community Council Directive 86/609, OJ L 358, 1, December 12, 1987; Italian Legislative Decree 116/92, Gazzetta Ufficiale della Repubblica ltaliana n. 40, February 18, 1992; National Institutes of Health Guide for the Care and Use of Laboratory Animals, National Institutes of Health Publication n. 85-23, 1985). All the procedures were approved by the Institutional Review Board of the Fondazione IRCCS Istituto Nazionale dei Tumori.
Study design	- Number of experimental groups: upon subcutaneous injection of human cancer cell lines, mice were divided into 4 cohorts of 8-10 mice per cohort and treated with vehicle, dasatinib, CHOEP or dasatinib+CHOEP.<br>- Experimental unit: 5- to 7-week-old female NOD.CB17-Prkdc^{scid}/J mice.
Experimental procedures	- Human cell lines injection: 15×10^{7} cells (HD-MAR-2 or OCI-Ly12) in 50% Matrigel were subcutaneously injected into the flanks of mice.<br>- Treatment: when tumor volume reached 150-200 mm^{3}, mice were randomly divided into 4 cohorts and treated as follows: control cohort: 0.2 ml of saline i.v. e 0.2 ml of H2O per OS. Dasatinib (DA) cohort: 30 mg/kg per OS, 5 days for 2 weeks. CHOEP cohort: one 5-days single cycle of CHOEP. Cyclophosphamide day 1, 40mg/kg i.v.; doxorubicin day 1, 3.3 mg/kg i.v.; vincristine day 1, 0.5 mg/kg i.v.; prednisone from day 1 to day 5, 0.2 mg/kg per OS; etoposide day 1 and day 3, 3.3 mg/kg i.v. DA+CHOEP cohort: cyclophosphamide day 1, 40mg/kg i.v.; doxorubicin day 1, 3.3 mg/kg i.v.; vincristine day 1, 0.5 mg/kg i.v.; prednisone from day 1 to day 5, 0.2 mg/kg per OS; etoposide day 1 and day 3, 3.3 mg/kg i.v., dasatinib from day 1 to day 5 and from day 8 to day 12, 30 mg/kg per OS. Cyclophosphamide, doxorubicin, vincristine and etoposide were diluted in saline and intravenously injected. Prednisone and dasatinib were diluted in water and administered per OS using an oral gavage.<br>- Weighing: body weight and tumor size were monitored before and after treatment, every 2-3 days.<br>- Euthanasia: at the end of the experiments, mice were euthanized by cervical dislocation.
Experimental animals	5- to 7-week-old female NOD.CB17-Prkdc^{scid}/J mice (Charles River), weight 15-25g.
Housing and husbandry	- Facility: standard facility, rooms with automatic systems of temperature, humidity and light regulation (temperature: 20-24°C; dark/light cycle: 12/12h; humidity: 60±5%).<br>- Cage: plastic, sterile, with filter.<br>- Cage companions, max 4 animals/cage.<br>- Bedding material: high adsorbing bedding material without dust, changed every week.<br>- Environmental enrichment was done with sterile material.
Sample size	OCI-Ly12-injected mice per cohort: ctrl n=8, DA n=8, CHOEP n=7, DA+CHOEP n=9.<br>HD-MAR-2-injected mice per cohort: ctrl n=8, DA n=8, CHOEP n=8, DA+CHOEP n=8.<br>Sample size was calculated using G*power software.
Allocating animals to experimental groups	Mice were divided into 4 experimental groups after randomization.

Experimental outcomes	To evaluate the antitumor activity of the combination dasatinib+CHOEP in *in vivo* models of T-cell lymphoma.
Statistical methods	Statistical analyses were performed with Two-way ANOVA test. Comparisons were performed using Bonferroni's multiple comparison test. The differences were considered significant at $P<0.05$.
